# Adaptive Compaction Construction Simulation Based on Bayesian Field Theory

**DOI:** 10.3390/s20185178

**Published:** 2020-09-10

**Authors:** Jun Zhang, Jia Yu, Tao Guan, Jiajun Wang, Dawei Tong, Binping Wu

**Affiliations:** State Key Laboratory of Hydraulic Engineering Simulation and Safety, Tianjin University, Tianjin 300000, China; zhangjunchn@tju.edu.cn (J.Z.); tao.guan@tju.edu.cn (T.G.); jiajun_2014_bs@tju.edu.cn (J.W.); tongdw@tju.edu.cn (D.T.); wubinping@tju.edu.cn (B.W.)

**Keywords:** adaptive compaction construction simulation, Bayesian field theory, simulation end condition, discrete event simulation

## Abstract

The compaction construction process is a critical operation in civil engineering projects. By establishing a construction simulation model, the compaction duration can be predicted to assist construction management. Existing studies have achieved adaptive modelling of input parameters from a Bayesian inference perspective, but usually assume the model as parametric distribution. Few studies adopt the nonparametric distribution to achieve robust inference, but still need to manually set hyper-parameters. In addition, the condition of when the roller stops moving ignores the impact of randomness of roller movement. In this paper, a new adaptive compaction construction simulation method is presented. The Bayesian field theory is innovatively adopted for input parameter adaptive modelling. Next, whether rollers have offset enough distance is used to determine the moment of stopping. Simulation experiments of the compaction process of a high earth dam project are demonstrated. The results indicate that the Bayesian field theory performs well in terms of accuracy and efficiency. When the size of roller speed dataset is 787,490, the Bayesian field theory costs only 1.54 s. The mean absolute error of predicted compaction duration reduces significantly with improved judgment condition. The proposed method can contribute to project resource planning, particularly in a high-frequency construction monitoring environment.

## 1. Introduction

The compaction construction operation is widespread in the construction of civil engineering projects and is a crucial process for ensuring project quality [1,2]. It can be described as that after spreading paving material, such as soil, gravel, and asphalt into a layer with a specific thickness. Load, together with vibration energy, is applied to the filling layer by rollers to make the layer compaction quality meet design requirements. The accurate prediction of the compaction duration can contribute to effective construction organization management and resource planning. The discrete event simulation (DES) method, as a popular tool in the civil engineering construction field [3,4,5,6,7], can achieve operation duration prediction by simulating the detailed resource flow based on the concept of entity and event. When building a construction simulation model, both the input parameter model and state transition conditions of resources are essential prerequisites and have a significant impact on the reliability of the model [8,9,10].

Over the past 20 years, advanced data sensing technology and communication technology [11] have been adopted for measuring, detecting, and tracking infrastructure [12], construction equipment [13,14], site personnel [15], and construction environment [16] during the construction process. Consequently, the capability of collecting samples of construction parameters is dramatically promoted. In contrast to traditional empirical assumptions about the input parameters, building a data-driven construction simulation model [10,17,18] based on the in situ construction monitoring system is becoming more popular. In existing data-driven construction simulation studies, when modeling the input parameters, the probability distributions are often built to reflect the inherent uncertainty of the construction process. Referring to the mathematics of probability theory, the input parameter modeling belongs to the statistical inference task of the probability distribution function. Besides, the adaptive modeling of input parameters is defined as an effective updating parameter model aligned with new arriving monitoring data to reflect the changing construction environment. Consequently, compared with the classical statistical inference method, the Bayesian statistical inference method [19] that, utilizing hypothesis prior information which expresses the degree of belief and new samples to compute the posterior probability distribution via Bayes’ rule, is more suitable. Under the Bayesian probabilistic framework, Chung T.H. et al. [20] firstly applied the Bayesian updating technique to update the uniform distribution of TBM penetration rate, TBM repair time, survey time, and the exponential distribution of TBM breakdown time, as an input to tunnel the TBM construction simulation model based on Simphony environment. Song L.G. and Eldin N.N. [21] proposed an adaptive real-time simulation framework for asphalt hauling and paving operations, in which the BestFit software provides fitting functions of 28 different probability distributions. Zhang S.R. et al. [22] proposed a construction simulation model to calculate the completion probability of the underground cavern group project. The prior of drilling machine production rate, loading machine production rate, transporting rock velocity, and returning velocity is assumed to be a normal distribution. Guan T. et al. [23] adopted the fuzzy set theory and normal distribution to describe the simulation parameters, and the improved resampling method is used to promote the representativeness of sample data during the Bayesian updating process. In the above studies, the prior distribution of input parameter of the simulation model is set as a predefined parametric distribution, which may bring a mismatch between model and data. Instead, in the Bayesian nonparametric statistical inference [24], the prior probability distribution is defined in infinite-dimensional function space, which can provide more robust inference compared to the parametric model with a specific functional form [25]. For a more detailed elaboration of the main classes of Bayesian nonparametric model, the studies of Gershman S.J. and Blei D.M. [26] and Mueller P et al. [27] are recommended. Recently, Hu W. et al. [28] adopted the Dirichlet process mixture model to represent the probability distribution of construction simulation parameters. However, the hyper-parameters still need to be manually set.

In this paper, to overcome these shortcomings without losing the powerful flexibility of the nonparametric model, the Bayesian field theory is innovatively introduced into the construction simulation field for adaptive modeling of input parameters. The Bayesian field theory approach [29] is a novel method for inferring potentially probability density distribution under low-dimensional scenario based on concepts of statistical physics. In the initial study, Bialek W et al. [30] defined a one-dimensional free scalar field with a constraint to reparametrize each candidate of the probability distribution function to be inferred. Nemenman I. and Bialek W. [31] proved that, if a prior of the potential probability density distribution is defined and represented as a linear combination of scale-dependent priors, the posterior distribution of the specified length scale parameter will peak sharply in the big data limit. Consequently, an alternative to cross-validation for selecting the optimal length scale parameter is presented. On the basis of Nemenman Ilya and Bialek William’s study, Kinney J.B. [32] proposed a method for naturally calculating the optimal length scale parameter from input data, which makes the application of scale-free field prior possible and computable. Based on the previous studies, Kinney J.B. [33] further unified the Bayesian field theory and maximum entropy estimation method to overcome the deficiency of scale-free Bayesian field theory that imposing boundary conditions on candidate densities. In the latest research, Chen W.C. et al. [29] adopted the importance resampling to correct the Laplace approximation applied to a large data regime. After reducing the non-Gaussian effects, Chen W.C. et al. [29] proposed a Bayesian field theory approach, which is simultaneously suitable for a small dataset. In general, unlike the common nonparametric classical statistical inference method, such as the kernel density estimation method, and the common nonparametric Bayesian statistical inference method, such as the Dirichlet process mixture modeling method, the Bayesian field theory approach does not require the manual identifying of values of critical parameters, specifying of boundary conditions, or making of invalid mathematical approximations in the small data regime, while realizing the optimal estimation [29].

In another aspect, defining involved events is the basis for establishing simulation logic when building the construction simulation model. If the state of construction machinery changes, it means the beginning and end of different construction events. Therefore, it is necessary to clearly define the state transition conditions of construction machinery. Existing compaction construction simulation research is mainly in the dam construction field and is summarized. Zhong D.H. et al. [34] built a high rockfill dam construction simulation model by integrating the haulage and placement subsystem and adopting the CYCLONE method. Based on this study, Zhang J. et al. [35] proposed a simulation model considering the adverse impact of stochastic rainfall on schedule by combing a modified daily stochastic precipitation generator. Du R.X. et al. [36] realized the comprehensive multi-objective simulation optimization of schedule, cost, and filling equilibrium degree based on DES, entropy weight method, and genetic algorithm. Zhang J et al. [37] further proposed an improved simulation optimization method by utilizing D-AHP and whale optimization algorithm. In the above research, the compaction construction process is simplified as a process with a certain duration. That is, the compaction construction process is treated as an event that does not involve a change of state. Zhong D.H. and Zhao C.S. [38] proposed a refined compaction construction simulation model for the first time from the point of view of simulating the movement of rollers. On this basis, Hu W. et al. [28] proposed a construction phase-oriented dynamic simulation method for a roller compacted concrete dam placement project. However, as an important member of the state transition conditions, the condition of when the roller stops moving is set as to whether a fixed offset times (or a fixed number of compaction bands) has been reached. For ease of expression, this condition is called the “simulation end condition”. The impact of the randomness of roller movement on compaction duration is ignored. In this paper, an improved simulation end condition matching the actual behavior of roller is proposed and set as to whether rollers have offset enough distance to consider the randomness of offset times.

By improving the two aspects, including the input parameter modeling method, and the state transition conditions of roller, a new adaptive compaction construction simulation method is proposed in this paper. The application potential of the Bayesian field theory method under real construction scenarios and big data conditions is explored for the first time. The rest of this paper is organized as follows. In Section 2, details of the proposed method are given, including the adaptive parameter modeling method and the improved compaction construction simulation method. In Section 3, experimental results are presented to verify the validity of the proposed method. In Section 4, more comparative analyses are discussed to further highlight the advantages of the proposed approach. In Section 5, the main conclusions are summarized.

## 2. Research Methodology

**Figure 1:**
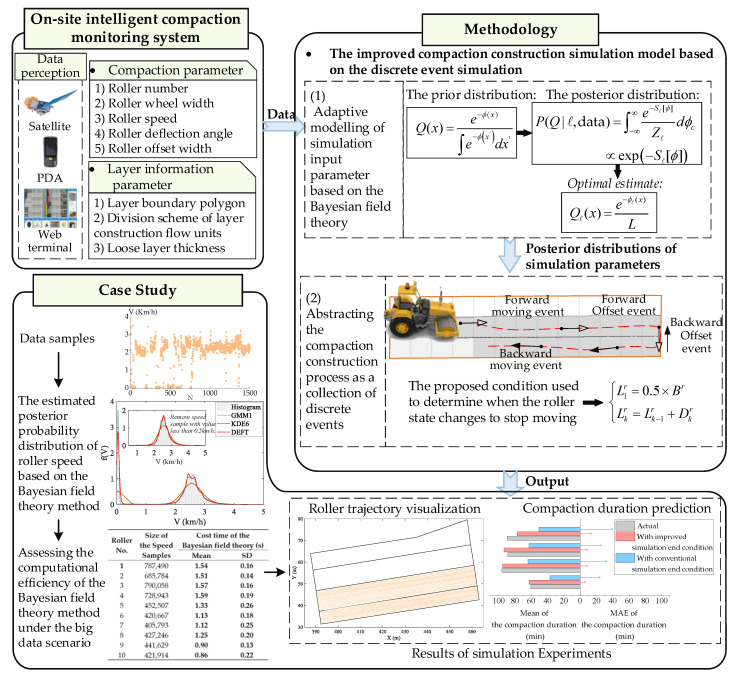
The overall framework of the proposed adaptive compaction construction simulation method.

In this study, a new adaptive compaction construction simulation method was proposed. The overall framework is shown in Figure 1. First, the necessary data need to be collected for adaptive modelling of the simulation input parameter. Through integrating satellite positioning technology, vibration monitoring technology and other sensing devices, such as accelerometers, the in situ intelligent compaction monitoring system for dam projects developed by the author’s research group [39,40,41] can realize continuous real-time monitoring of the compaction construction process. Here the GPS positioning data of rollers is mainly used. The roller trajectory is composed of continuous positioning points and can be further used to extract samples of roller deflection angle and roller offset width. The layer information parameters, such as layer boundary and layer division scheme, are also recorded into the database. The input parameter model is an important component of the compaction construction simulation model. After collecting the monitoring data, the Bayesian field theory is innovatively adopted to provide the posterior probability distribution of the simulation parameter close to the changing construction scene without predefined hyper-parameters. Finally, an improved compaction construction simulation model based on discrete event simulation is proposed to implement schedule forecasting of the compaction process. To establish a rational compaction construction simulation model, the compaction process is regarded as the reciprocating moving process of rollers and is further abstracted as a discrete collection of rollers forward, backward, forward offset and backward offset events. On the basis of the defined deflection angle, the updating equations of roller position in different construction events are built. In order to determine when the compaction construction is over—that is, when the roller state changes to stop moving—the condition is defined as whether rollers have offset adequate distance. In contrast to previous studies, the randomness of the demand offset times due to the combined effect of the roller deflection angle and roller offset width is considered. The proposed compaction construction simulation model can realize the visual simulation of roller moving trajectory and thus enables the prediction of compaction duration. In the following sections, the proposed method is described in detail.

### 2.1. Adaptive Modeling of Simulation Input Parameter Based on Bayesian Field Theory

This section shows how to accomplish adaptive modeling of the probability distribution of the simulation input parameter based on the Bayesian field theory method. Suppose that X is a random variable representing a simulation input parameter, and ${\left\{x_{i}\right\}}_{i=1}^{N}$ are the data points of the variable X collected by the on-site intelligent compaction monitoring system. Assuming that ${\left\{x_{i}\right\}}_{i=1}^{N}$ is independent and identically distributed, the modeling task is to obtain the one-dimensional probability distribution function Q(x) of X based on the latest sample collection ${\left\{x_{i}\right\}}_{i=1}^{N}$.

According to Chen W. C. et al.’s study [28], the latest research of the Bayesian field theory approach, ϕ (−∞<ϕ<∞) as a defined real scalar field variable with a constraint is firstly adopted to reparametrize and normalize each candidate of Q(x). Assume that the Bayesian prior distribution of Q(x) is denoted as P[Q(x)] and can be abbreviated as P(Q). P(Q) is further defined as a linear combination of scale-dependent priors P(Q|ℓ) to make the calculation truly feasible. Here ℓ is an arbitrary smoothness length scale that controls the gradient penalty degree applied to Q(x), and will affect the hypothesized smoothness of Q(x). The mathematical expression of Q(x), P(Q), and the prior P(ϕ|ℓ) of ϕ can be, respectively, written as:

(1) $$Q(x)=\frac{e^{-\phi (x)}}{\int e^{-\phi \left(x^{\prime }\right)}dx^{\prime }}$$

(2) $$P(Q)=\int_{0}^{\infty }P(Q|\ell )P(\ell )d\ell$$

(3) $$P(\phi |\ell )=\frac{e^{-S_{\ell }^{0}[\phi ]}}{Z_{\ell }^{0}}$$

where $S_{\ell }^{0}[\phi ]=\int \frac{dx}{L}\frac{\ell^{2\alpha }}{2}{\left(\partial^{\alpha }\phi \right)}^{2}$ is the prior action after introducing the Fourier representation, L is a length factor that controls the normalization degree of Q(x), α is the derivation order and is an arbitrary positive integer, $Z_{\ell }^{0}=\int D\phi e^{-S_{\ell }^{0}[\phi ]}$ is the corresponding prior partition function.

Combining Equations (1) and (3), the prior P(Q|ℓ) can be expressed as:

(4) $$P(Q|\ell )=\int_{-\infty }^{\infty }p(\phi |\ell )d\phi_{c}=\sqrt{\frac{2\pi }{\epsilon }}\frac{e^{-S_{\ell }^{0}\left[\phi_{nc}\right]}}{Z_{\ell }^{0}}$$

where $\phi (x)=\phi_{nc}(x)+\phi_{c}$, $\phi_{nc}(x)$ and $\phi_{c}$ are the constant Fourier component and non-constant Fourier component of ϕ(x), respectively.

Given the independent identically distributed property, P(data|Q) is the likelihood of Q and can be expressed as:

(5) $$\begin{array}{l} P(data|Q)=P(x_{1},x_{2},\ldots ,x_{N}|Q(x))\\ \;=\prod_{n=1}^{N}Q\left(x_{n}\right)\\ \;=\frac{N^{N}}{L^{N}\Gamma (N)}\int_{-\infty }^{\infty }e^{-\int \frac{dx}{L}\left\{N*L*R*\phi +N*e^{-\phi }\right\}}d\phi_{c} \end{array}$$

where N is the total number of samples.

Combining Equations (4) and (5), P(data,Q|ℓ) and the posterior action $S_{\ell }[\phi ]$ can be shown as:

(6) $$P(data,Q|\ell )=\frac{N^{N}}{L^{N}\Gamma (N)}\sqrt{\frac{2\pi }{\epsilon }}\frac{1}{Z_{\ell }^{0}}\int_{-\infty }^{\infty }e^{-S_{L}[\phi ]}d\phi_{c}$$

(7) $$S_{\ell }[\phi ]=\int \frac{1}{L}\left(\frac{\ell^{2\alpha }}{2}{\left(\partial^{\alpha }\phi \right)}^{2}+N*L*R*\phi +N*e^{-\phi }\right)dx$$

where $R(x)=N^{-1}\sum_{i=1}^{N}\delta \left(x-x_{i}\right)$ is the histogram summarizing the raw data probability density.

The evidence P(data|ℓ) can be expressed as:

(8) $$P(\mathrm{data}|\ell )=\frac{N^{N}}{L^{N}\Gamma (N)}\sqrt{\frac{2\pi }{\epsilon }}\frac{Z_{\ell }}{Z_{\ell }^{0}}$$

where the $Z_{\ell }$ is the posterior partition function and can be expressed as $Z_{\ell }=\int D\phi e^{-S_{l}[\phi ]}$.

According to the Bayesian theorem, combining Equations (6) and (8), the posterior P(Q|ℓ,data) can be calculated as:

(9) $$\begin{array}{l} P(Q|\ell ,\mathrm{data})=\frac{P(\mathrm{data},Q|\ell )}{P(\mathrm{data}|\ell )}\\ \;=\int_{-\infty }^{\infty }\frac{e^{-S_{\ell }[\phi ]}}{Z_{\ell }}d\phi_{c}\\ \;\propto \exp\left(-S_{\ell }[\phi ]\right) \end{array}$$

In Chen W.C. et al.’s study [28], the importance resampling method is utilized during the sampling process of P(Q|ℓ,data). Based on the maximum posterior density estimation (MAP) method and Equation (9), the optimal estimate $Q^{*}$ of Q(x) can be obtained when the following is satisfied:

(10) $$\delta S_{\ell }[\phi ]/\delta \phi =0$$

The value of ϕ that minimizes $S_{\ell }[\phi ]$ is assumed to be $\phi_{\ell }$, the solution of Equation (10) satisfies:

(11) $$\ell^{2\alpha }\Delta^{\alpha }\phi_{\ell }+NLR-Ne^{-\phi_{\ell }}=0$$

Corresponding optimal estimation $Q_{\ell }(x)$ is:

(12) $$Q_{\ell }(x)=\frac{e^{-\phi_{\ell }(x)}}{L}$$

During the calculation, for sets of the smoothness length scale $\left\{\ell_{0},\ell_{1},\ell_{2},\ldots ,\ell_{K}\right\},\ell_{0}=0\sim \ell_{K}=\infty$, the optimal value of the smoothness length scale $\ell^{*}$ can be chosen through maximizing the Bayesian belief P(data|ℓ). After determining $\ell^{*}$, the optimal probability distribution function $Q^{*}$ can be uniquely determined, according to Equation (12). Here the $Q^{*}$ is the target probability distribution of the simulation input parameter that needs to be calculated.

### 2.2. The Improved Compaction Construction Simulation Model Based on Discrete Event Simulation

**Figure 2:**
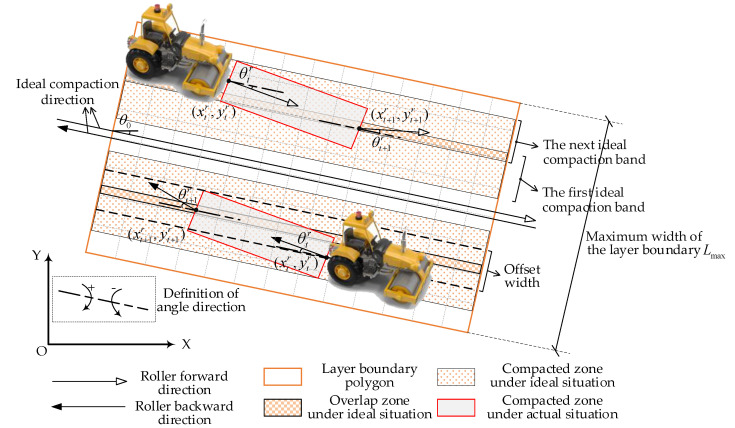
Overview of a filling layer.

In order to build the logic of the compaction construction simulation model, the core lies in defining the involved construction events and the state transition conditions of rollers. For each construction event, the position updating equations of rollers need to be defined as the basis of the simulation model. Figure 2 shows a filling layer to be simulated, where the O−XY axis is the defined coordinate system. The boundary shape of the filling layer is represented as a polygon. In the practical construction scenario, the area to be constructed is generally divided into several flowing units to reduce total construction time and then assigned to multiple idle rollers.

Due to the influence of driving behavior and uneven layer surface, the movement of the roller shows certain randomness. This causes the actual compacted areas to be out of alignment with the design compaction direction, as shown in Figure 2. In this paper, the compaction process is regarded as the reciprocating movement of several rollers under the coupling effect of random roller speed and random roller deflection angle, and is further abstracted as a discrete collection of rollers forward, backward, forward offset and backward offset events. The offset event refers to that after completing two unidirectional movements on the first compaction band, the roller offsets a certain width (defined as the offset width) to one side, and then continues the compaction of the next band.

For establishing the updating equations of roller position coordinates, two angles are defined. As shown in Figure 2, the $\theta_{0}$ represents the angle between the ideal moving direction of a roller and the positive X-axis, and the deflection angle $\theta^{r}$ describes the angle between the random forward or backward direction of the roller and the ideal moving direction. If $\theta_{0}>0$, it indicates that the ideal moving direction is clockwise on positive X-axis. If $\theta_{r}>0$, it suggests that the actual moving direction is clockwise on the ideal moving direction. When simulating the roller forward and backward events, the roller speed $v_{t}^{r}$ and the roller deflection angle $\theta_{t}^{r}$ at t moment are drawn from their corresponding distribution. Assuming that the roller r’s positioning coordinate is $\left(x_{t}^{r},y_{t}^{r}\right)$ at t moment, then the roller positioning coordinate $\left(x_{t+1}^{r},y_{t+1}^{r}\right)$ at t+1 moment under forwarding moving condition and backward moving condition can be, respectively, updated through Equations (13) and (14).

(13) $$\mathrm{If}\;x_{t}^{r}>x_{t-1}^{r}:\{\begin{array}{l} x_{t+1}^{r}=x_{t}^{r}+v_{t}^{r}\cos\left(\theta_{0}+\theta_{t}^{r}\right)\\ y_{t+1}^{r}=y_{t}^{r}-v_{t}^{r}\sin\left(\theta_{0}+\theta_{t}^{r}\right) \end{array}$$

(14) $$\;\mathrm{If}\;x_{t}^{r}\leq x_{t-1}^{r}:\{\begin{array}{l} x_{t+1}^{r}=x_{t}^{r}-v_{t}^{r}\cos\left(\theta_{0}+\theta_{t}^{r}\right)\\ y_{t+1}^{r}=y_{t}^{r}+v_{t}^{r}\sin\left(\theta_{0}+\theta_{t}^{r}\right) \end{array}$$

If the roller moves twice on one compaction band and reaches the layer boundary, it will offset a random width to one side. When simulating the offset event, the kth offset width $D_{k}^{r}$ of the roller r at t moment is also taken from its probability distribution function. Accordingly, the updating equations of the roller position coordinate $\left(x_{t+1}^{r},y_{t+1}^{r}\right)$ in forwarding offset event and backward offset event are, respectively, shown in Equations (15) and (16).

(15) $$\;\mathrm{If}\;x_{t}^{r}>x_{t-1}^{r}:\{\begin{array}{l} x_{t+1}^{r}=x_{t}^{r}-D_{k}^{r}\sin\theta_{0}\\ y_{t+1}^{r}=y_{t}^{r}+D_{k}^{r}\cos\theta_{0} \end{array}$$

(16) $$\;\mathrm{If}\;x_{t}^{r}\leq x_{t-1}^{r}:\{\begin{array}{l} x_{t+1}^{r}=x_{t}^{r}+D_{t}^{r}\sin\theta_{0}\\ y_{t+1}^{r}=y_{t}^{r}+D_{t}^{r}\cos\theta_{0} \end{array}$$

If the roller completes the random walk in its feasible construction zone, the roller state will change to stop moving and the compaction process ends. In previous studies, the fixed offset times or the fixed number of compaction bands are adopted to determine when the compaction construction simulation ends. However, the uncertainty of offset times under the combined effect of random deflection angle and random offset distance is ignored. In this study, an improved simulation end condition is proposed. As shown in Figure 2, for a filling layer, the width to be completed $L_{\max}$ is defined as the maximum width of the layer boundary shape perpendicular to the ideal moving direction. Assuming that the wheel width of roller r is $B^{r}$, after the kth offset, the completed width $L_{k}^{r}$ is calculated by the Equation (17). When the completed width $L_{k}^{r}$ is not less than the maximum width $L_{\max}$ and the roller position coordinate is outside the layer boundary, the simulation of the initial compaction process is finished.

(17) $$\left\{ \begin{array}{l} L_{1}^{r}=0.5\times B^{r}\\ L_{k}^{r}=L_{k-1}^{r}+D_{k}^{r} \end{array}\right.$$

**Figure 3:**
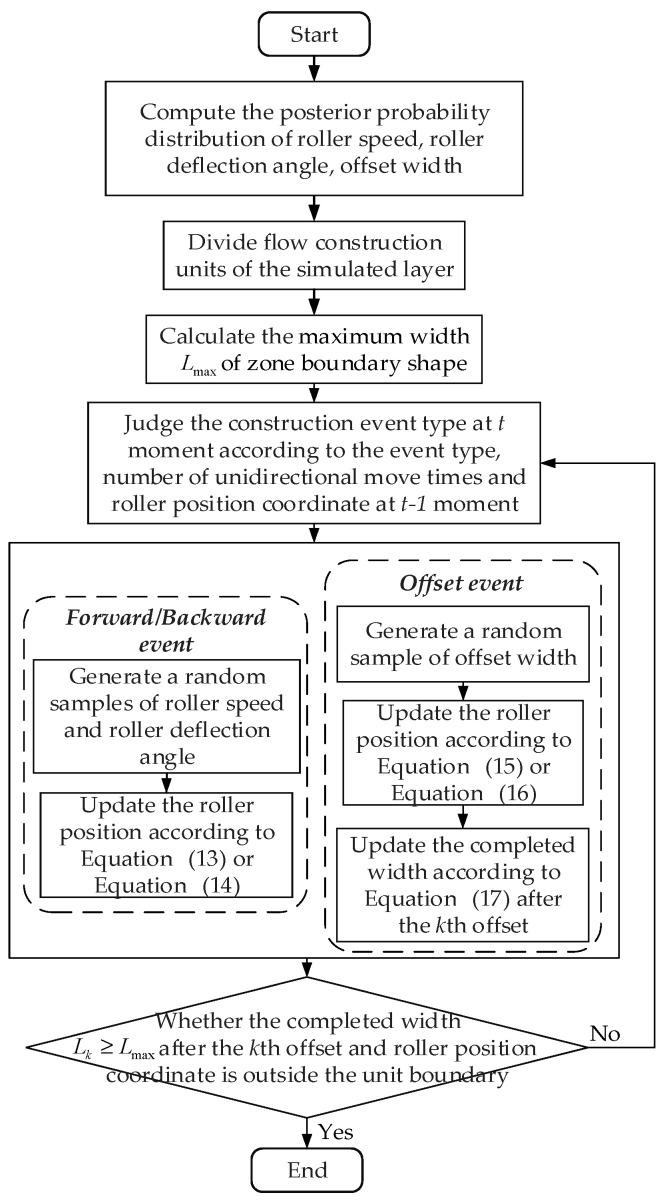
Flowchart of the proposed adaptive compaction construction simulation model.

The total flow chart of the proposed adaptive compaction construction simulation model is shown in Figure 3, in which the detailed simulation logic and steps are presented.

## 3. Case Study

The compaction construction process was the key link for ensuring dam construction quality and project safety. To verify the validity of the proposed method in this paper, construction simulation experiments were conducted on the compaction process of a high earth dam project in Southwest China. The case study was divided into two parts. Firstly, the distribution modeling of roller speed was realized based on the Bayesian field theory method. As a key input parameter of the compaction construction simulation model, the roller speed greatly affects the compaction duration. Then, taking the result distributions as input, the compaction duration prediction was realized based on the improved compaction construction simulation model. In the practical construction process, filling layers were often divided into several flow units to reduce the duration. Here the simulated layer was divided into four flow units, each with one roller for compaction operation. The basic information of each flow unit is shown in Table 1. The field datasets for modelling simulation input parameters were collected from the on-site intelligent compaction monitoring system developed by the author’s research group. The new arriving data were collected every second, and the time period was from September 1st, 2018 to September 30th, 2018. Experiments were all run on a computer of Intel i7-6820HQ 2.70GHz CPU, 16.0 GB RAM, Windows 10 System.

### 3.1. Adaptive Modeling of the Roller Speed Based on Bayesian Field Theory

**Figure 4:**
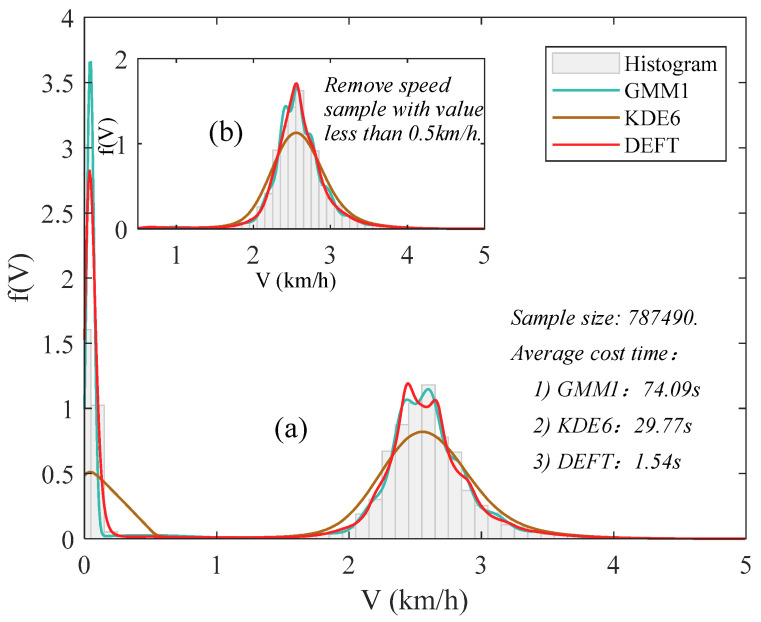
The fitted distributions of the speed of roller 1 in September 2018. GMM1 refers the optimal Gaussian mixture model based on Akaike’s Information Criterion [42]; KDE6 refers the kernel density estimation method with linear kernel [43]; DEFT refers to the Bayesian field theory method.

Take the roller 1 as an example, in September 2018, the sample size of roller speed dataset collected by the on-site intelligent compaction construction monitoring system was 787,490. Based on the Bayesian field theory method, the fitted distributions of the speed of roller 1 are exhibited in Figure 4. For ease of elaboration, refer to J.B. Kinney’s study (2014). The Bayesian field theory method for probability density estimation is abbreviated as DEFT (Density Estimation using Field Theory). The Gaussian mixture model [42] and the kernel density estimation method [43] are adopted for comparison. Specifically, the optimal number of Gaussian distribution components in the Gaussian mixture model was determined by the Akaike’s Information Criterion. The kernel function of the kernel density estimation method was set as the linear kernel. The two methods were abbreviated as GMM1 and KDE6, respectively. It is clearly shown in Figure 4a that, in terms of fitting accuracy, the Bayesian field theory method works best when compared to the Gaussian mixture model and the kernel density estimation method. Moreover, the probability density is close to 0 when the speed is under 0.22 km/h. During the actual compaction construction process, when the roller speed is lower than a specific threshold value, it often means that the roller is in the initial startup stage, and the corresponding data sample should be eliminated when modeling. Here 0.5 km/h was set as the threshold to enlarge the difference between normal samples and abnormal samples. After excluding the value below 0.5 km/h, the sample size of the new dataset was 572,476. The new probability distribution of the speed of roller 1 is shown in Figure 4b. It shows a unimodal characteristic, and the probability density achieves a peak value when the speed value is 2.56 km/h.

**Figure 5:**
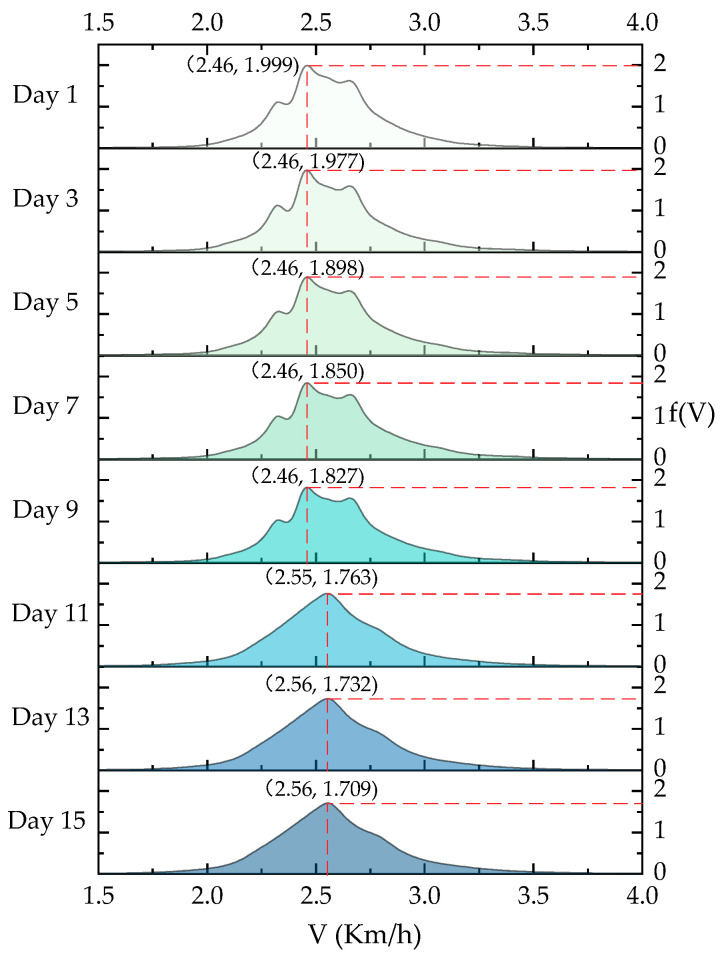
The updated posterior distributions of the speed of roller 1 from September 1, 2018, to September 15, 2018.

For visually showing the impact of continuous arriving monitoring data, two days was set as the update time step. The estimated Bayesian posterior distributions of the speed of roller 1 from September 1 to September 15 are illustrated in Figure 5. Taking Day 15 as an example (September 15), the coordinates (2.56, 1.709) indicate that the probability density reaches a peak value of 1.709 when the roller speed takes the value 2.56 km/h. As shown in Figure 5, for roller 1, the updating speed distribution gradually approaches the final distribution shown in Figure 4 and becomes stable on September 13th.

### 3.2. Compaction Construction Simulation Results of a Layer in a High Earth Dam Project

**Figure 6:**
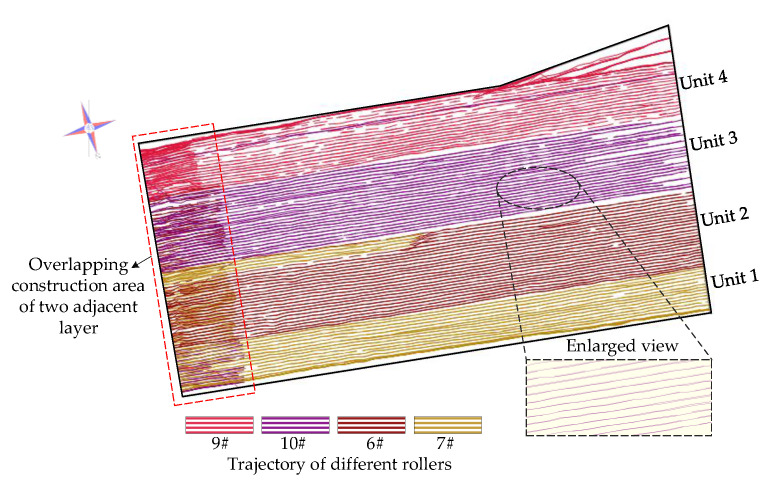
The actual roller trajectory of the simulated layer obtained from the intelligent compaction construction monitoring system.

**Figure 7:**
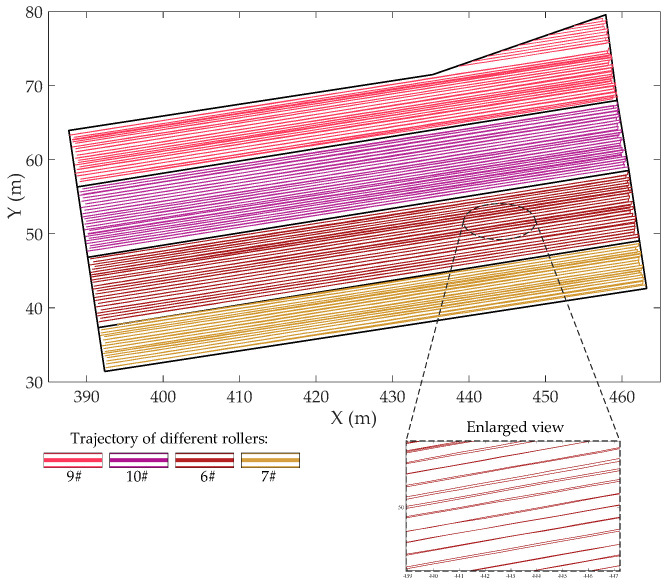
The simulated roller trajectory of the simulated layer obtained from the proposed compaction construction simulation model.

Given the characteristic of Bayesian posterior distribution of roller speed (after excluding sample value less than 0.5 km/h), the normal distribution was adopted as reference distribution, and then the rejecting sampling method was used to obtain random samples from inferred probability distributions during the simulation process. To visually demonstrate the effectiveness of the improved compaction construction simulation method proposed in this paper, different from the previous research, the actual roller trajectory of the simulated layer obtained from the intelligent compaction construction monitoring system and the simulated roller trajectory are presented in Figure 6 and Figure 7 for visual comparison.

In Figure 6, the polygon composed of black lines is the geometric boundary of the simulated layer, and curves of different colors represent the actual trajectory of different rollers. It should be noted that the area in the red box on the left side is the overlapping construction area of two adjacent layers. The case study here is conducted on only one layer to mitigate the impact of the short work stoppage, so there is no overlapping area in the simulated roller trajectory. As shown in Figure 7, the simulation trajectory is made up of folded lines and effectively reproduces the characteristics of the actual trajectory.

After 50 repeated simulation experiments, the compaction duration results obtained from the monitoring system and simulation experiments are shown in Table 2. For the simulated units 1 to 4, the mean absolute error indexes (MAE) of the predicted compaction duration are 5.75 min, 8.23 min, 8.59 min, and 13.13 min, which are 9.47%, 8.56%, 9.68%, and 17.13% of the actual values, respectively. The values of the MAE index prove the effectiveness of the proposed modeling method of the simulation input parameter and the proposed compaction construction simulation method. Furthermore, the effects of different modeling methods of the simulation input parameter can be seen in Table 2. The low local accuracy of the GMM1 and KDE6 method results in more prediction errors of the compaction duration.

## 4. Discussions

To further demonstrate the advantages of the approach presented in this paper, multiple comparative analyses were carried out and are detailed in this section.

### 4.1. Evaluating the Computing Accuracy of the Bayesian Field Theory Method

Artificial datasets generated from known probability distribution are considered to quantitatively verify the accuracy of the Bayesian field theory method on the probability distribution inference task. For distinguishing the effects of normal characteristics, a mixed normal distribution $f(x_{1})$ and a non-normal mixed distribution $f(x_{2})$ were adopted to generate artificial datasets:

(18) $$f(x_{1})=0.25\times N(-1,0.5)+0.4\times N(1,0.6)+0.35\times N(2.5,0.4)$$

(19) $$f(x_{2})=0.3\times N(-1.5,0.3)+0.5\times U(-0.5,1.5)+0.2\times N(2.5,0.4)$$

Based on $f(x_{1})$ shown in Equation (18), 50 random sample sets of size 100 were generated. Based on $f(x_{2})$ shown in Equation (19), 50 random sample sets of size 1000, 5000, and 10,000 were separately generated.

The current state-of-the-art methods were selected for comparison, including the Gaussian mixture model (GMM) in the field of classical parametric statistical inference, the kernel density estimation method (KDE) in the field of classical nonparametric statistical inference, and the Dirichlet process mixture model (DPMM) in the field of nonparametric Bayesian statistical inference. The GMM model uses the Expectation-Maximization algorithm (EM) to determine the optimal number of Gaussian distribution components in the range of [1,10] based on Akaike’s Information Criterion and Bayesian Information Criterion, respectively. In order to reduce the influence of the subjective selection of kernel function type, the KDE method adopts six different kernel functions, including the Gaussian kernel, the Tophat kernel, the Epanechnikov kernel, the Exponential kernel, the Cosine kernel, and the Linear kernel. The bandwidths were all set as 0.5. The DPMM model adopts the Gaussian distribution as the base distribution. Abbreviations of other methods used for comparison are shown in Table 3.

When finishing estimating, the Jensen–Shannon divergence (JSD) is used as the evaluation metric to measure the difference between the estimated probability density distribution and the true probability density distribution. The JSD overcomes two deficiencies of the frequently used Kullback–Leibler Divergence (KLD), including asymmetric and unboundedness. Expression of the JSD is shown in Equation (20), the smaller the JSD value, the closer the estimated probability density distribution to the true probability density distribution.

(20) $$\left\{ \begin{array}{l} KLD(f,g)=\int f(x)\log\left\{\frac{f(x)}{g(x)}\right\}dx\\ JSD(f(x)||g(x))=\frac{1}{2}KLD(f(x)||\frac{f(x)+g(x)}{2})+\frac{1}{2}KLD(g(x)||\frac{f(x)+g(x)}{2}) \end{array}\right.$$

**Figure 8:**
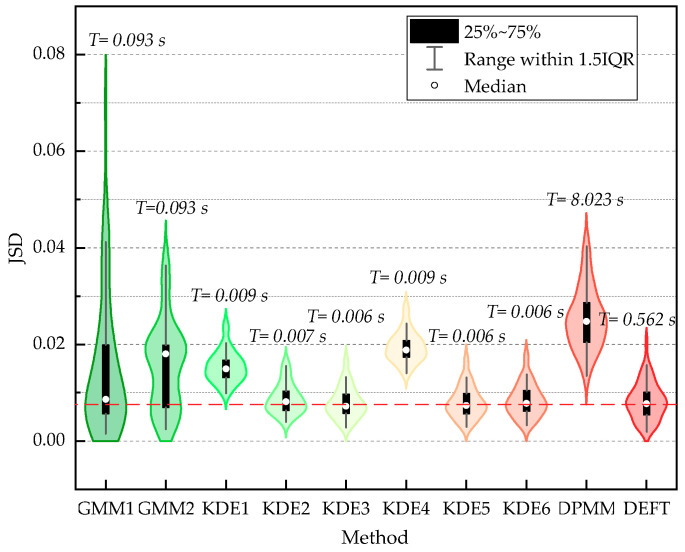
The JSD and average cost time results of fitting 50 random datasets of size 100 generated from $f(x_{1})$.

**Figure 9:**
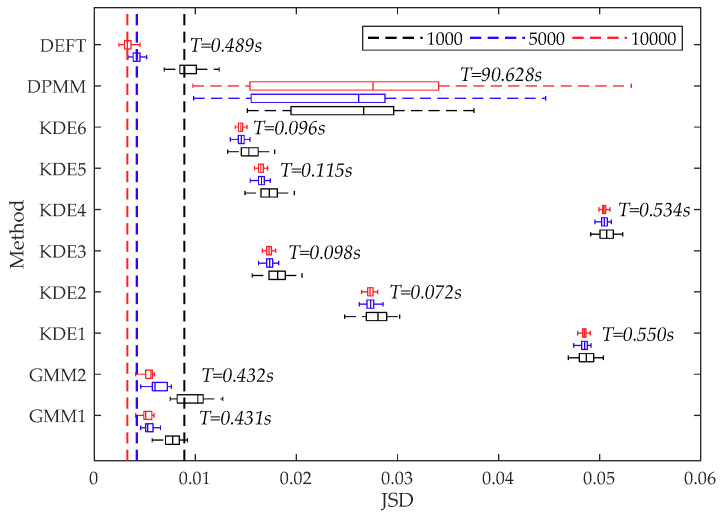
The JSD results of fitting 50 random datasets of size 1000, 5000, 10,000 generated from $f(x_{2})$T represents the average cost time of fitting 50 random datasets of size 10,000.

**Figure 10:**
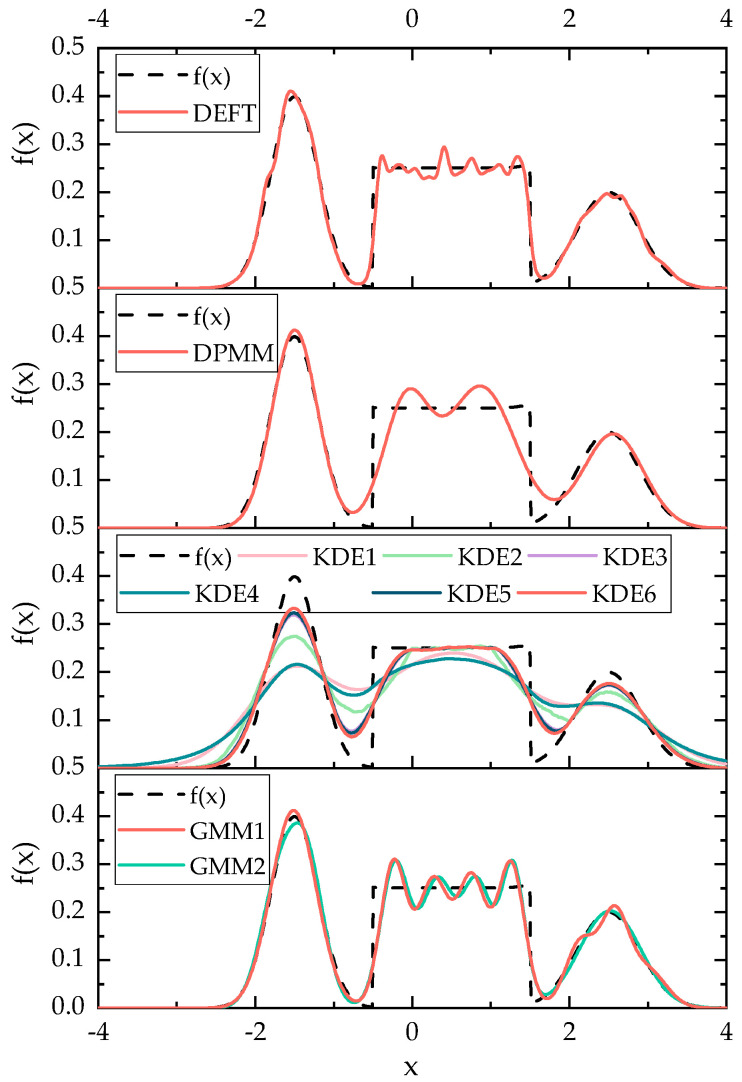
The fitted distributions of a random dataset of size 10,000 generated from $f(x_{2})$.

The JSD and average cost time results of fitting 50 random datasets of size 100 generated from $f(x_{1})$ are displayed in Figure 8. The JSD results of fitting 50 random datasets of size 1000, 5000, 10,000 generated from $f(x_{2})$, and the average calculation time when the sample size was 10,000 are displayed in Figure 9. In addition, when the sample size was 10,000, a single calculation result for visual comparison is shown in Figure 10.

As shown in Figure 8 and Figure 9, the JSD measure highlights that the GMM1, the KDE6, and the DEFT provide the best estimation on all artificial datasets generated from $f(x_{1})$ and $f(x_{2})$. The descending order of fitting accuracy is DEFT > GMM1 > KDE6. In addition, the box plot confirms the computing stability of the DEFT method. As shown in Figure 10, the fitted density curve shows that the DEFT provides a satisfactory estimate closest to the real probability distribution. For the middle trough part, the KDE6 provides a very poor estimate, and the GMM1 is not effective. Moreover, for the middle non-normal probability densities, the GMM1 method provides a very poor estimate. This reflects the shortcomings brought by predefined distribution types in the parametric model.

In summary, in terms of computing accuracy, the Bayesian field theory method performs well on the probability distribution inference task for both normal and non-normal distributions.

### 4.2. Evaluating the Computing Efficiency of the Bayesian Field Theory Method

In this section, the speed datasets of rollers collected by the intelligent compaction construction monitoring system are adopted to evaluate the computing efficiency of the Bayesian field theory method. The description of the size of ten roller speed datasets is listed in Table 4. After 50 times of repeated simulation experiments, the mean and standard deviation of cost time of GMM1, KDE6, and DEFT method are summarized in Table 4. Since the DPMM takes much longer than 24 h when the dataset size is 787,490, it is not included in Table 4.

In Section 4.1, as shown in Figure 8 and Figure 9, when fitting 50 random datasets of size 100 generated from $f(x_{1})$, the average cost time of the GMM1, the KDE6, the DPMM, and the DEFT is 0.093 s, 0.006 s, 8.023 s, and 0.562 s, respectively. When fitting 50 random datasets of size 10,000 generated from $f(x_{2})$, the average cost time of the GMM1, the KDE6, the DPMM, and the DEFT is 0.431 s, 0.096 s, 90.628 s, and 0.489 s, respectively. It is worth noting that as the sample size increases, the cost time of the GMM1 and KDE6 obviously increases, the cost time of the DPMM increases sharply, while the cost time of the DEFT is almost unchanged. Different from the artificial datasets, the speed dataset is much larger. As shown in Table 4, when the size of the speed dataset is within 405,793 to 452,507, the cost time of the DEFT is within 0.86 s to 1.33 s. When the size of the speed dataset is within 685,784 to 790,058, the cost time of the DEFT is within 1.51 s to 1.59 s, and increases less than one second. Compared with GMM1 and KDE6, DEFT still has a sizeable computational efficiency advantage for large-scale high-frequency monitoring data. In addition, the small SD proves that the cost time of DEFT is stable. To sum up, the above experiments reveal that, in terms of accuracy and computational efficiency, the Bayesian field theory shows obvious superiority in addressing the probability distribution inference task, and is much more suitable for the adaptive modeling of the input parameter of the compaction construction simulation model, especially under the high-frequency construction monitoring environment.

### 4.3. Analyzing the Effectiveness of Improved Simulation End Condition

**Figure 11:**
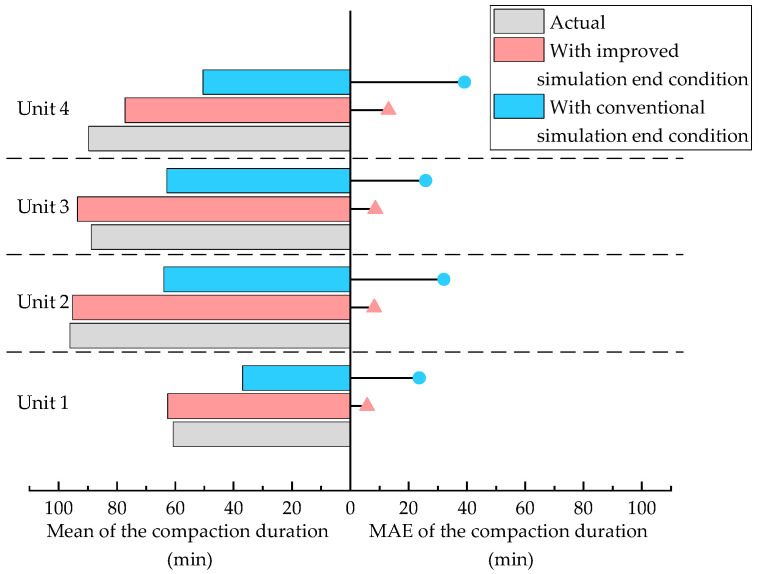
Comparison of the Mean and MAE of the compaction duration of each unit under different simulation end conditions.

To verify the effectiveness of the improved simulation end condition proposed in this paper, the compaction construction simulation experiments with conventional simulation end condition are conducted. The conventional simulation end condition refers to the use of a fixed offset times or a fixed number of compaction bands. After 50 repeated simulation experiments, the mean, standard deviation and mean average value of predicted compaction duration results are presented in Figure 11. Compared to the results in Table 2, the MAEs of the predicted compaction duration increase from 5.75 min, 8.23 min, 8.59 min, 13.13 min to 23.73 min, 32.18 min, 25.88 min, 39.17 min. It can be illustrated that the prediction accuracy of compaction duration improves significantly with the proposed simulation end condition. By visualizing the moving trajectory of rollers, it can be seen that rollers do not complete the compaction construction process of their designated area under the conventional simulation end condition. The premature stopping of the construction simulation of roller moving process results in a significantly smaller predicted compaction duration.

## 5. Conclusions

The compaction construction operation is a common and key process in civil engineering projects. The prediction of compaction duration is helpful to the reasonable formulation of the whole project schedule. Aiming at the shortcomings in existing compaction construction simulation researches, including lack of flexibility and the need to manually set hyper-parameters when modelling simulation input parameters, and ignoring the impact of the randomness of roller movement when defining the simulation end condition, a new adaptive compaction construction simulation method that enables more accurate compaction duration prediction is proposed in this paper. The simulation experiments on four filling units of a high earth dam project in Southwest China demonstrate the validity of the proposed method in this paper. The main contributions of this article are: (1) The novel Bayesian field theory method is introduced into the construction simulation field for the first time. The irreplaceable advantages and the application potential of the Bayesian field theory method on the simulation parameter modelling tasks are highlighted. (2) An improved simulation end condition is presented to consider the randomness of the offset action of rollers, and the corresponding prediction accuracy increases significantly. The method proposed in this paper provides a means to achieve compaction duration prediction and resource planning of the complicated construction process, especially for the high-frequency construction monitoring environment. There is still further work that can be done to improve the proposed method. During the computational analysis, it was found that the shapes of roller trajectory are very sensitive to the mean and standard deviation of the roller deflection angle. In the next step, to further explore how to build a more reasonable compaction construction simulation model, sensitivity analysis can be used to quantitatively evaluate the impact degree of roller deflection angles on compaction duration. In addition, building a benchmark dataset for better testing and comparison of construction simulation models is meaningful and deserves consideration in future research.

## Figures and Tables

**Table 1:** Basic information about the four simulated flow units.

Flow Unit No.	Area of the Unit (m^2^)	Arranged Roller No.
1	447.8	9
2	688.1	10
3	688.1	6
4	591.9	7

**Table 2:** Compaction duration results of the simulated layer after replicating the simulation experiment 50 times with improved simulation end condition.

Unit No.	Actual Compaction Duration (min)	Predicted Compaction Duration (min)									
		GMM1			KDE6			DEFT			
		Mean	SD	MAE	Mean	SD	MAE	Mean	SD	MAE	
1	60.67	**61.41**	**7.13**	5.82	61.91	8.53	6.64	62.65	8.27	**5.75**	
2	96.10	94.86	11.30	9.50	94.13	**9.59**	7.99	**95.20**	11.93	**8.23**	
3	88.75	97.90	12.62	11.10	96.67	10.23	9.08	**93.61**	**10.18**	**8.59**	
4	89.68	76.64	7.93	13.78	**78.11**	9.86	**13.10**	77.19	**7.80**	13.13	

The numbers in bold represent the optimal Mean, SD, and MAE.

**Table 3:** The ten probability distribution inference methods for comparative analysis.

Number	Inference Method	Description	Abbreviation
1	Gaussian Mixture model [42]	AIC information criterion	GMM1
2		BIC information criterion	GMM2
3	Kernel density estimation [42]	Gaussian kernel	KDE1
4		Tophat kernel	KDE2
5		Epanechnikov kernel	KDE3
6		Exponential kernel	KDE4
7		Cosine kernel	KDE5
8		Linear kernel	KDE6
9	Dirichlet process mixture model [43]	Gaussian base distribution	DPMM
10	Bayesian field theory	/	DEFT

**Table 4:** The mean and standard deviation of the cost time of different methods on the probability distribution inference task of ten roller speed datasets.

Roller No.	Size of the Speed Samples	Cost Time (s)					
		GMM1		KDE6		DEFT	
		Mean	SD	Mean	SD	Mean	SD
1	787,490	74.09	6.58	29.77	2.77	**1.54**	**0.16**
2	685,784	68.94	2.82	26.59	0.81	**1.51**	**0.14**
3	790,058	101.23	10.11	34.83	1.31	**1.57**	**0.16**
4	728,943	78.28	3.30	29.37	2.29	**1.59**	**0.19**
5	452,507	57.98	3.95	12.99	0.94	**1.33**	**0.26**
6	420,667	42.80	2.27	17.63	1.37	**1.13**	**0.18**
7	405,793	39.54	4.01	13.77	0.71	**1.12**	**0.25**
8	427,246	50.32	3.91	11.49	0.99	**1.25**	**0.20**
9	441,629	53.21	4.75	13.85	0.90	**0.90**	**0.13**
10	421,914	43.95	5.14	13.41	0.78	**0.86**	**0.22**

Numbers in bold indicate the smallest mean and standard deviation of cost time.

## References

[1] Liu D., Li Z., Lian Z. (2014). Compaction quality assessment of earth-rock dam materials using roller-integrated compaction monitoring technology. Autom. Constr..

[2] Wang J., Zhong D.-H., Adeli H., Wang N., Liu M. (2018). Smart bacteria-foraging algorithm-based customized kernel support vector regression and enhanced probabilistic neural network for compaction quality assessment and control of earth-rock dam. Expert Syst..

[3] Abourizk S. (2010). Role of Simulation in Construction Engineering and Management. J. Constr. Eng. Manag..

[4] Shawki K., Kilani K., Gomaa M. (2015). Analysis of earth-moving systems using discrete-event simulation. Alex. Eng. J..

[5] Goh Y.M., Ali M.J.A. (2016). A hybrid simulation approach for integrating safety behavior into construction planning: An earthmoving case study. Accid. Anal. Prev..

[6] Bokor O., Florez L., Osborne A., Gledson B.J. (2019). Overview of construction simulation approaches to model construction processes. Organ. Technol. Manag. Constr. Int. J..

[7] Krantz J., Feng K., Larsson J., Olofsson T. (2019). ‘Eco-Hauling’ principles to reduce carbon emissions and the costs of earthmoving—A case study. J. Clean. Prod..

[8] Akhavian R., Behzadan A.H. (2015). Construction equipment activity recognition for simulation input modeling using mobile sensors and machine learning classifiers. Adv. Eng. Inform..

[9] Louis J., Dunston P.S. (2017). Methodology for Real-Time Monitoring of Construction Operations Using Finite State Machines and Discrete-Event Operation Models. J. Constr. Eng. Manag..

[10] Shrestha P., Behzadan A.H. (2018). Chaos Theory–Inspired Evolutionary Method to Refine Imperfect Sensor Data for Data-Driven Construction Simulation. J. Constr. Eng. Manag..

[11] Yang J., Park M.-W., Vela P.A., Golparvar-Fard M. (2015). Construction performance monitoring via still images, time-lapse photos, and video streams: Now, tomorrow, and the future. Adv. Eng. Inform..

[12] Du L., Zhong R., Sun H., Zhu Q., Zhang Z. (2018). Study of the Integration of the CNU-TS-1 Mobile Tunnel Monitoring System. Sensors.

[13] Alshibani A., Moselhi O. (2016). Productivity based method for forecasting cost & time of earthmoving operations using sampling GPS data. J. Inf. Technol. Constr..

[14] Kim H., Bang S., Jeong H., Ham Y., Kim H. (2018). Analyzing context and productivity of tunnel earthmoving processes using imaging and simulation. Autom. Constr..

[15] Mehrang S., Pietilä J., Korhonen I. (2018). An Activity Recognition Framework Deploying the Random Forest Classifier and a Single Optical Heart Rate Monitoring and Triaxial Accelerometer Wrist-Band. Sensors.

[16] Cheung W.-F., Lin T.-H., Lin Y.-C. (2018). A Real-Time Construction Safety Monitoring System for Hazardous Gas Integrating Wireless Sensor Network and Building Information Modeling Technologies. Sensors.

[17] Akhavian R., Behzadan A.H. (2013). Knowledge-Based Simulation Modeling of Construction Fleet Operations Using Multimodal-Process Data Mining. J. Constr. Eng. Manag..

[18] Vahdatikhaki F., Hammad A. (2014). Framework for near real-time simulation of earthmoving projects using location tracking technologies. Autom. Constr..

[19] Bolstad W.M. (2007). Introduction to Bayesian Statistics.

[20] Chung T.H., Mohamed Y., Abourizk S. (2006). Bayesian Updating Application into Simulation in the North Edmonton Sanitary Trunk Tunnel Project. J. Constr. Eng. Manag..

[21] Song L., Eldin N.N. (2012). Adaptive real-time tracking and simulation of heavy construction operations for look-ahead scheduling. Autom. Constr..

[22] Zhang S., Du C., Sa W., Wang C., Wang G. (2014). Bayesian-Based Hybrid Simulation Approach to Project Completion Forecasting for Underground Construction. J. Constr. Eng. Manag..

[23] Guan T., Zhong D.-H., Ren B.-Y., Song W.-S., Chu Z.-Q. (2018). Construction simulation of high arch dams based on fuzzy Bayesian updating algorithm. J. Zhejiang Univ. Sci. A.

[24] Ghosal S., Van Der Vaart A. (2017). Fundamentals of Nonparametric Bayesian Inference.

[25] MacEachern S.N. (2016). Nonparametric Bayesian methods: A gentle introduction and overview. Commun. Stat. Appl. Methods.

[26] Gershman S.J., Blei D.M. (2012). A tutorial on Bayesian nonparametric models. J. Math. Psychol..

[27] Mueller P., Quintana F.A., Page G. (2017). Nonparametric Bayesian inference in applications. J. Ital. Stat. Soc..

[28] Hu W., Zhong D., Wu B., Li Z. (2019). Construction phase oriented dynamic simulation: Taking RCC dam placement process as an example. J. Civ. Eng. Manag..

[29] Chen W.-C., Tareen A., Kinney J.B. (2018). Density Estimation on Small Data Sets. Phys. Rev. Lett..

[30] Bialek W., Callan C.G., Strong S.P. (1996). Field Theories for Learning Probability Distributions. Phys. Rev. Lett..

[31] Nemenman I., Bialek W. (2002). Occam factors and model independent Bayesian learning of continuous distributions. Phys. Rev. E.

[32] Kinney J.B. (2014). Estimation of probability densities using scale-free field theories. Phys. Rev. E.

[33] Kinney J.B. (2015). Unification of field theory and maximum entropy methods for learning probability densities. Phys. Rev. E.

[34] Zhong D.-H., Zhang P., Wu K. (2007). Theory and practice of construction simulation for high rockfill dam. Sci. China Ser. E Technol. Sci..

[35] Zhang J., Zhong D.-H., Wu B.-P., Lv F., Cui B. (2017). Earth Dam Construction Simulation Considering Stochastic Rainfall Impact. Comput. Civ. Infrastruct. Eng..

[36] Du R., Zhong D.-H., Yu J., Tong D., Wu B.-P. (2016). Construction Simulation for a Core Rockfill Dam Based on Optimal Construction Stages and Zones: Case Study. J. Comput. Civ. Eng..

[37] Zhang J., Zhong D.-H., Zhao M., Yu J., Lv F. (2019). An Optimization Model for Construction Stage and Zone Plans of Rockfill Dams Based on the Enhanced Whale Optimization Algorithm. Energies.

[38] Zhong D.H., Zhao C.S. (2012). Theory and application of construction simulation coupled with quality factors for high core rock-fill dam. Water Resour. Hydropower Eng..

[39] Zhong D.-H., Cui B., Liu D., Tong D. (2009). Theoretical research on construction quality real-time monitoring and system integration of core rockfill dam. Sci. China Ser. E Technol. Sci..

[40] Zhong D.-H., Liu D., Cui B. (2011). Real-time compaction quality monitoring of high core rockfill dam. Sci. China Ser. E Technol. Sci..

[41] Liu Y., Zhong D.-H., Cui B., Zhong G., Wei Y. (2015). Study on real-time construction quality monitoring of storehouse surfaces for RCC dams. Autom. Constr..

[42] Pedregosa F., Varoquaux G., Gramfort A., Michel V., Thirion B., Grisel O., Blondel M., Prettenhofer P., Weiss R., Dubourg V. (2011). Scikit-learn: Machine Learning in Python. JMLR.

[43] Gordon J.R., Dean M., Kees M. (2019). Dirichletprocess: Build Dirichlet Process Objects for Bayesian Modelling. R Package Version 0.3.1. https://CRAN.R-project.org/package=dirichletprocess.

